# Why Do We Watch? The Role of Emotion Gratifications and Individual Differences in Predicting Rewatchability and Movie Recommendation

**DOI:** 10.3390/bs10010008

**Published:** 2019-12-19

**Authors:** Patrícia Arriaga, Joana Alexandre, Octavian Postolache, Manuel J. Fonseca, Thibault Langlois, Teresa Chambel

**Affiliations:** 1 CIS-IUL, ISCTE-Instituto Universitário de Lisboa, 1649-026 Lisboa, Portugal; joana.alexandre@iscte-iul.pt; 2 IT-IUL, ISCTE-Instituto Universitário de Lisboa, 1649-026 Lisboa, Portugal; octavian.adrian.postolache@iscte-iul.pt; 3 LASIGE, Faculdade de Ciências, Universidade de Lisboa, 1749-016 Lisboa, Portugal; mjfonseca@ciencias.ulisboa.pt (M.J.F.); tl@di.fc.ul.pt (T.L.); mtchambel@ciencias.ulisboa.pt (T.C.)

**Keywords:** emotional gratifications, movie recommendation, rewatchability, extraversion, need for cognition, need for affect

## Abstract

Background: The present study’s main aim was to determine the predictors of movie rewatchability and recommendations. Methods: Using a sample of 318 participants, we first tested the structure of a gratification scale from watching a movie. Then, we examined the role of age, need for cognition, need for affect, extraversion, and emotional gratifications, in predicting individuals’ interest in rewatching the movie and in making recommendations. Results: As in the original proposal of the emotional gratification scale, the following dimensions were identified: fun, thrill, empathic sadness, release of emotions, social sharing, contemplative experiences, and character engagement, with acceptable model fit and reliability, convergent and divergent validity. Social sharing, contemplate experiences, need for affect and age were significant predictors of movie recommendation; whereas social sharing, thrill, extraversion, and age contributed most to explaining rewatching interest. Conclusion: This study highlights the importance of considering distinct gratifications and individual differences in predicting rewatching and movie recommendation.

## 1. Introduction

Several theories have been developed to explain why we watch movies and to predict the appeal of entertainment experiences. The gratification model [1] emphasizes the importance of emotions, either directly, by being gratifying experiences per se; or indirectly, by contributing to social and cognitive needs. Direct gratifications include experiencing positive states (e.g., fun) or negative (e.g., sadness) and mixed feelings (e.g., kindness) often associated with the appeal of tragic entertainment [2]. Regardless of the valence of emotions, it is also important to note the arousal or thrill the movies elicit [1]. Theoretical models, such as the mood management theory, highlighted the preference of individuals to select the type of movie that are expected to maintain or maximize pleasurable states and achieve optimal levels of arousal. Empathic models suggested that the empathy felt towards characters involved in negative situations may provide gratification because empathy is a morally valued feeling [1,2], which may explain the gratification paradox of negative feeling states. The second group of gratifications is related to social and cognitive needs [1]. Social gratifications include the need for sharing emotional experiences with others, with the media being a means to build and strengthen interpersonal relations [3,4,5]. Social gratifications can also involve the engagement felt with and towards the media characters through identification, by cultivating “parasocial interaction” with the characters, resembling real social relationships [6,7,8]. Additionally, the need for being stimulated cognitively by aesthetic experiences involving contemplativeness, challenging and/or meaningful experiences that fulfill individual needs of self-reflection, purpose in life, or self-development have been highlighted [9,10,11]. These eudaimonic motivations are central in models that distinguish reactive from reflective aesthetic experience [12], or hedonic from eudaimonic approaches of well-being [13], and may often include narratives that evoke communal feelings of being moved by seeing other’s affection [14,15,16]. Based on empirical studies and theories, Bartsch [1] has developed the Emotional Gratification Scale (EGS) identifying seven reasons of why media experience can be regarded as gratifying, which includes the above experiences of fun, thrill, empathic sadness, contemplative experiences, social sharing, character engagement, and vicarious release of emotions. This last factor emerged from interviews and corresponds to the need in releasing, through the media, the emotions avoided in everyday life. 

Individual differences may also contribute to explain entertainment use, such as the need for affect (NFA), the need for cognition (NFC), and extraversion. NFA is a general motivation to approach or avoid situations/stimuli that elicit strong emotions. Individuals with stronger NFA tend to be more emotionally responsive to the media content, by reporting higher levels of emotions [17]. NFC is intrinsically a motivation to elaborate and prefer cognitive challenges, thought-provoking and complex narratives [18], and like NFA, it has been found to predict eudaimonic gratifications from entertainment [10,19,20]. 

Extraversion is a personality dimension that includes facets such as gregariousness, seeking for excitement, and positive affect, and it has been related to the experience of both positive [21] and ambivalent affects with movies [17]. Regarding age, there has been inconsistent results, except that older participants tend to report more gratification from movies that provide contemplative experiences [1]. 

Less studies have been conducted to understand why some individuals watch the same content repeatedly. Nowadays, rewatching becomes easy to achieve, given the advances in digital technologies and networking, allowing individuals to access movies in a more affordable way (e.g., streaming the media through television channels or websites). Bentley and Murray [22] conducted interviews and a survey to understand the reasons underlying the motivation in rewatching movies and television series. The main reasons were similar to the gratifications outlined by Bartsch [1]. Social sharing emerged as the main reason for rewatching. Additionally, frequently reported was the interest in parasocial interaction with the characters, and the motivation to feel desirable emotional states (e.g., to have fun, feel excitement). Participants also indicated their interest in rewatching complex stories to understand more deeply the narrative and/or view details that they might have missed before. Finally, younger individuals seemed to find more enjoyment in rewatching movies [22].

Another important indicator of interest in a movie is whether an individual recommends it to other people. Nowadays, it is possible for many individuals to express opinions, interests, and make recommendations through the internet to a broader community of online users. Consequently, many online rating systems have been developed, which also gather users’ ratings or detailed reviews, assuming that the consumer’s information reflects their level of satisfaction about the product. Based on these systems, many studies are using collaborative filtering methods to estimate users’ patterns of interests and then make recommendations, by considering users’ reviews and preferences [23]. Studies have highlighted the importance of viewers’ recommendations of a movie [24], but to the best of our knowledge, no study has investigated the role of a viewer’s emotional gratifications in predicting recommendation, besides the role of mood state at the time of evaluation [25] or the identification of emotions expressed by users in movie reviews to map its emotional content [26]. 

Based on prior theories and empirical findings, we included in our study specific emotional gratifications from movies and individual differences that are expected to make relevant contributions to predict movie recommendation and rewatching. Since these variables have not been tested in conjunction, we followed an exploratory approach. Since the EGS was originally validated in a distinct language, we also examined the latent structure of the scale. Based on the original validation [1], we hypothesized a 7-factor model, and expected acceptable fit, reliability and validity indexes.

## 2. Materials and Methods

All procedures followed the 2013 Declaration of Helsinki ethical standards for research and were approved by the University ethical committee in which the study took place (REF63/19). Using a cross-sectional design, participants were selected by a non-probability sampling procedure, and recruited via convenience and snowball sampling methods. Students enrolled in a Psychology graduation course of a Portuguese University, where the convenience initial sample took place, were then asked to collaborate in collecting the data among their friends and acquaintances.

After providing the informed consent, participants were given a paper and pencil survey that included the following instruments:

The Emotional Gratification Scale (EGS) [1], which was followed by an instruction requiring participants to think of a movie they liked and remembered well. After naming the movie title, participants evaluated, in each of the EGS’s 28 items, the reasons to choose the movie, distributed evenly by seven dimensions (four items each): contemplative experiences, fun, thrill, character engagement, vicarious release of emotions, empathic sadness, and social sharing of emotions, on a 5-point scale (1 = totally disagree; 5 = totally agree). The English and the Portuguese translated items can be found in Appendix A (Table A1). For cross-cultural adaptation to the Portuguese language, two researchers translated independently the instrument to Portuguese by making adequate semantic changes to allow equivalence, which was then back translated by an independent bilingual person. The translations were then discussed until a consensus was reached. 

Movie Recommendation was measured with the question, “How much would you recommend this movie to a friend?” and Rewatching interest with the question, “How much would you like to see this movie again?”; both answered on a 5-point scale (1 = *Never*; 5 = *Very Much*). 

The preferences for complexity was measured using the Need for Cognition (NFC) scale [18,27], by applying only the five items of the Portuguese version for the Complexity subscale because it relates to the individual’s preferences for being cognitively stimulated from the environment (e.g., *I would prefer complex to simple problems*). Responses were given on a 5-point scale (1 = *I do not identify myself*; 5 = *I strongly identify myself*). In our study, Cronbach’s α = 0.83. The Portuguese version of the Need for Affect (NFA) questionnaire [28,29] was used to measure the approach dimension, because this subscale has been found to predict viewers’ responses to movies [17]. This subscale has five items (e.g., *I feel that I need to experience strong emotions regularly*), answered on a 7-point scale, ranging from −3 (*strongly disagree*) to 3 (*strongly agree*), with α = 0.76. The Portuguese version of the 12 items of the extraversion dimension taken from the NEO-Five Factor Inventory [NEO-FFI] [30,31] was answered on a 5-point scale ranging from 0 (*strongly disagree)* to 4 (*strongly agree*) (α = 0.70). Finally, sociodemographic information (e.g., age, gender) was collected, with other measures unrelated to the present report. 

## 3. Results

### 3.1. Descriptive Statistics

From the original sample (*n* = 329), 11 participants were excluded for not responding on age (2.4%) and all items of the scales (0.9%). The remaining 318 (200 women; 118 men; age 18–65, *M* = 27.87; *SD* = 11.28) were used in the analyses. Regarding a participant’s nationality, the majority were Portuguese (95.6%), and the remaining reported being Brazilian, Canadian, Mozambican, or Santomense.

As the scales presented some Missing Completely at Random (Little’s MCAR tests *ps* > 0.05), the expectation–maximization method was used to replace missing values. The size of 318 is adequate for the confirmatory factor analysis (CFA) (ratio of 11 participants per item), and sufficient for the multiple linear regression analysis (MLRA), based on a priori sample size estimation indicating that a minimum of 179 participants would be required, considering conventional Cohen’s medium effect sizes (*f*^2^ = 0.15), power of 0.95, *p* < 0.05, and maximum of 11 predictors.

### 3.2. Structure of the Emotional Gratifications Scale

To analyze the EGS latent structure, we conducted CFAs with maximum likelihood estimation using the IBM SPSS AMOS 24.0 software. The data was normally distributed (Multivariate Kurtosis < 1.4). We allowed the items to only load on a single factor, and the factors to correlate. Based on recommendations [32], we report the following indexes: the χ2/df and the Standardized Root Mean Square Residual (SRMR) for the Absolute fit, the Comparative Fit Index (CFI) for the Incremental fit, and the Root Mean Square Error of Approximation (RMSEA) for Parsimony-Adjusted fit. The following fit thresholds are considered acceptable: 1 < χ2/df < 3, SRMR < 0.08, CFI > 0.90, and RMSEA < 0.08 [33,34]. 

In our first CFA, we found that the 7-factor model with 28 items did not sufficiently fit the data in our sample (χ2/df = 3.34, CFI = 0.85, SRMR = 0.093, RMSEA = 0.09, 90% CI 0.080–0.092). To improve the goodness-of-fit we considered several indicators (e.g., magnitude of the loadings in each factor; presence of cross-loadings; large modification indexes (MI); large standardized expected parameter change (SEPC), and standardized residuals) [35]. As three items presented cross-loadings (i.e., high correlations with items from a different factor) and lower factor weights (< 0.64), they were removed through an iterative process from the following dimensions: empathic sadness (“I like to be overwhelmed with emotion”), contemplative experiences (“inspired new insights”), and character engagement (“I identified with the characters’ outlook on life”). The item of sadness presented a lower loading (0.42), cross-loading (stronger correlation with items from thrill than with its own dimension), and a residual covariance with absolute value >2.58 with nine other items. The other two items also showed high standardized residual with four items. 

Table 1 presents both convergent and divergent validity results and factor loadings for the Emotional Gratification Scale with 25 items. 

The decision to remove these three items led to acceptable fit indexes: χ2/df=2.65, CFI = 0.90, SRMR = 0.07, RMSEA = 0.07, 90% CI 0.07–0.08, convergent validity (composite reliability = 0.73–0.91), and divergent validity (i.e., Maximum Shared Variance < Average Variance Extracted (AVE); square root of AVE > inter-construct correlations) [36]. EGS also presented internal consistency (0.73 < α < 0.91). Except for fun, only associated positively with thrill (0.13) and character engagement (0.29), and negatively with contemplative (−0.21), all the other factors presented positive correlations, ranging from 0.20 (thrill with contemplative) and 0.52 (thrill and character engagement). Overall, a similar 7-factor model was identified in our post-hoc model.

### 3.3. Predictors of Movie Recommendation and Interest in Rewatching Movies

Correlations between the individual differences and the gratifications showed low but statistically significant relations. Age was correlated with contemplative (0.14) and emotional release (0.12); NFC with contemplative experiences (0.18) and thrill (0.16); NFA with contemplative (0.27), character engagement (0.24), sadness (0.17), thrill (0.16), and social sharing (0.13); and extraversion with thrill (0.18) and fun (0.17), all *p* < 0.05. The correlation between rewatching and recommendation was 0.54, *p* < 0.001, indicating that although related, they are distinct constructs. Therefore, both measures were not aggregated in a single factor, and two MLRA were conducted separately. 

Table 2 presents descriptive, zero-order correlations, and the MLRA results. In each MLRA we only included the predictors with significant correlations with the outcomes. The MLRA includes the following coefficients: (i) standardized beta (β) indicating each predictor contribution holding the other predictors constant; (ii) general dominance weights (Dom) addressing multicollinearity, allowing to rank each predictor weight, and determining if a predictor contributes more variance than other predictors to the model; and (iii) commonality, showing the variance in the outcomes that are uniquely (U) and commonly (C) explained by predictors. Dom and commonality coefficients were included because β estimates are unstable when predictors are correlated [37]. The MLRA results were computed using the R software (version 3.5.1) [37]. For recommendation, vicarious release and fun were the only variables not included in the MLRA; for rewatching we included age, extraversion, NFA, thrill, social sharing, character engagement, and contemplative emotional experiences. 

As can be seen in Table 2, the Dom weights showed that social sharing dominated the contribution to regressions for both outcomes, compared to the other predictors. Based on the Dom comparison between each pair of predictors and significant β, we identified the following rank order of predictors that contributed with most incremental variance in predicting movie recommendation: social sharing, contemplative experiences, age, and NFA. The model explained 14% of movie recommendation variance. Commonalities indicated that the unique contribution of each predictor was in total 6%. For rewatching interest, the model explained 13% of the variance, with individual predictors alone contributing with a similar 6% of the variance. Social sharing contributed with the most unique variance to this outcome. Dom weight indicated that social sharing, thrill, extraversion, and age, were the most relevant predictors. The remaining were not statistically significant, and their unique contribution was negligible, after controlling for the other predictors.

## 4. Discussion and Conclusion

The main aim of this study was the identification of the type of gratifications and individual differences that predict individuals’ interest in rewatching and in recommending the movie they liked and recalled. We also tested the EGS factor structure based on the 7-factor model [1]. By eliminating three items showing cross-loadings, we found acceptable model fit and reliable indexes, as well as convergent and divergent validity, consistent with the theoretical constructs [1]. Thus, our study contributed to the examination of an instrument that allows the measurement of gratifications from movies of viewers in Lusophone countries. 

Emotional gratifications seem to play an important role in explaining the appeal of popular media, including movies and television series [1,12]. Many of the gratifications were above the mean point of the scale (except for empathic sadness and emotional release), with higher scores for thrill, followed by contemplative, social sharing, character engagement, and fun. These results suggest that thrill, but also eudaimonic and social gratifications, were the main sources of gratifications with the movies chosen by the participants. 

Consistently, most gratifications were also positively correlated to our outcomes, except fun and emotional release, and empathic sadness for rewatching. These findings are in line with studies and theories highlighting that cognitive and social gratifications are more commonly reported than hedonic experiences of fun, empathic sadness, and emotional release [1,9,10,11,19]. 

The relationships found among the predictors were expected, such as the positive association among the individual differences (e.g., NFA and NFC) [10], and between these variables and gratifications (e.g., NFA and NFC with eudaimonic reasons) [10,20,38]; between age and contemplative [1], and also among the gratifications [1], including the negative association between fun and contemplative reasons, demonstrating the theoretical distinction between hedonic and eudaimonic gratifications [10,12]. 

Our study was also able to identify the factors that contributed most to predicting movie recommendation and interest in rewatching, while considering the unique and shared variance among predictors. Social sharing contributed most to explaining both outcomes, a result consistent with theories highlighting that social sharing is a strong human motivation to develop and maintain social bonds [3,4]. Recommending movies is a social act, consistent with the need to share social experiences. The association with rewatching is also consistent with prior work showing that sharing seems to be the main reason for rewatching [22]. Age also indicated that younger participants tend to report higher interest in rewatching [22] and more willingness in recommending the movie. The predictors of these two outcomes were distinct. Individuals with high NFA and who reported more contemplative reasons reported more willing to recommend the movie to others; whereas being more extravert and having felt more thrill with the movie were the additional factors explaining rewatching interest. Concerning the emotional gratifications, it was interesting to find that viewers were more willing to recommend the movie they considered evoking more contemplative experiences in them, which might be related to another social motivation: the attainment of status or credibility among others, a strong motivation in sharing information on social media [39]. This motivation was not addressed in our study and should be investigated in the future. Besides the social sharing needs, internal factors (e.g., thrill) were relevant to explain the individual’s own interest in repeating the movie experience. Concerning the individual differences, it is difficult to explain theoretically why the NFA only remained a predictor for movie recommendation but not for rewatching; and why extraversion only remained a significant predictor in explaining rewatching, after accounting for the other variables. Although these variables were found to be correlated to both outcomes, they do not contribute uniquely with a significant percentage, when accounting for the other predictors. Similar negligible contributions were found for NFC, empathic sadness, and character engagement, which were unexpected. 

Limitations include the correlational design and not allowing causal inferences. Future studies should consider an experimental approach. The use of scales typically adds concerns, such as response bias difficult to overcome or control. However, as Bartsch [1] highlighted, the use of scales measuring an individual’s needs are “essential to the concept of emotional gratification, and should, therefore, be covered by an introspective measure” (p. 296). Nevertheless, these measures can be complemented by other methods such as physiological or think aloud protocols. Furthermore, the fact that our study was run in Portugal, using convenience and snowball sampling procedures, limits the generalization of our findings. We also highlight the exploratory nature of the present study, intended to generate hypotheses about the factors that predict movie recommendation and interest in rewatching a movie. Additionally, we recognize the low magnitude of the explained variance in both models. Although the explained variance is of similar magnitude found in previous studies [1], it suggests that additional determinants should be considered in future studies.

Despite these limitations, which should be addressed in future studies, our findings make additional contributions to the assessment of gratification and in understanding their role in movie recommendation and rewatching interest, but further research is also necessary to add more insights regarding the factors that contribute to predict our interest in movies. 

## Figures and Tables

**Table 1:** Validity (convergent and divergent) and factor loadings for the Emotional Gratification Scale.

Factors	α	CR	AVE	MSV	Loadings	Correlations						
						1	2	3	4	5	6	7
1. Fun	0.91	0.91	0.71	0.10	0.76–0.92	0.84 ^†^						
2. Contemplative	0.83	0.84	0.63	0.23	0.64–0.78	−0.21 ***	0.80 ^†^					
3. Thrill	0.73	0.77	0.46	0.45	0.80–0.90	0.13 *	0.20 **	0.68 ^†^				
4. Character	0.79	0.80	0.57	0.45	0.66–0.81	0.29 ***	0.39 ***	0.52 ***	0.76 ^†^			
5. Vicarious Release	0.85	0.85	0.59	0.29	0.70–0.82	−0.01	0.39 ***	0.30 ***	0.38 ***	0.77 ^†^		
6. Sadness	0.89	0.90	0.74	0.29	0.71–0.85	−0.004	0.43 ***	0.27 ***	0.33 ***	0.46 ***	0.86 ^†^	
7. Sharing	0.83	0.84	0.56	0.30	0.66–0.81	0.05	0.43 ***	0.45 ***	0.38 ***	0.26 ***	0.30 ***	0.75 ^†^

* *p* < 0.05, ** *p* < 0.01, *** *p* < 0.001; † average factor loadings; α *=* Cronbach’s alpha; CR = composite reliability; AVE = average variance extracted; MSV = maximum shared variance.

**Table 2:** Descriptive (means, standard deviation), Zero-Order Correlations, and Multiple Linear Regression Analyses for Movie Recommendation and Rewatchability.

Predictors	*M* (*SD*)	Recommendation					Rewatchability				
		*r*	β	Dom	U	C	*r*	β	Dom	U	C
Age	27.87 (11.28)	−0.15 *	−0.16 **	0.024	0.025	0.004	−0.13 *	−0.13 *	0.017	0.015	0.002
Need for cognition (range 1 to 5)	3.21 (0.87)	0.12 *	0.04	0.006	0.001	0.013	0.08	--	--	--	--
Extraversion (range 0 to 4)	2.56 (0.47)	0.13 *	0.03	0.006	0.001	0.016	0.18 **	0.12 *	0.018	0.011	0.021
Need for affect (range −3 to 3)	1.67 (0.92)	0.20 ***	0.12 *	0.020	0.011	0.029	0.11 *	0.02	0.004	0	0.012
Fun (range 1 to 5)	3.03 (1.25)	0.07	--	--	--	--	0.08	--	--	--	--
Thrill (range 1 to 5)	3.30 (0.97)	0.20 ***	0.07	0.014	0.003	0.036	0.20 ***	0.15 *	0.035	0.014	0.056
Empathic Sadness (range 1 to 5)	2.22 (1.21)	0.13 *	−0.01	0.004	0	0.018	0.07	--	--	--	--
Character (range 1 to 5)	3.11 (1.07)	0.19 ***	0.03	0.010	0	0.036	0.19 ***	0.02	0.011	0	0.035
Social Sharing (range 1 to 5)	3.17 (1.07)	0.25 ***	0.13 *	0.029	0.012	0.050	0.27 ***	0.16 *	0.038	0.018	0.053
Contemplative (range 1 to 5)	3.28 (1.21)	0.23 ***	0.13 *	0.024	0.011	0.041	0.14 *	0.04	0.007	0.001	0.018
Emotion Release (range 1 to 5)	2.38 (1.07)	−0.02	--	--	--	--	0.03	--	--	--	--
Model *R*^2^				0.14	0.06	0.24			0.13	0.06	0.20

* *p* < 0.05, ** *p* < 0.01, *** *p* < 0.001; *r* = zero-order correlation; *β* = standardized beta coefficients; Dom = general dominance weights; U = unique effects; C = common effects.

## Data Availability

The dataset of this study will be available from the corresponding author on request.

## Appendix A

Context of administration: After asking respondents to think of a movie they liked and remembered well, naming the title of the film, or briefly describing the story, participants are asked to evaluate the following 28 items of the EGS to the extent in which they have enjoyed the feelings associated with the movie, on a response scale that ranged from 1 (totally disagree) to 5 (totally agree).

**Table A1:** Emotional Gratification Scale [2]: English and Portuguese Items.

English Items	Portuguese Items
Fun	Diversão
because it was funny.	porque era divertido.
because it amused me.	porque divertiu-me.
because it made me laugh.	porque fez-me rir.
because it put me in a good mood.	porque deixou-me bem-humorado/a.
Contemplative experiences	Experiências contemplativas
because it made me think about myself.	porque fez-me pensar sobre mim próprio/a.
because it encouraged me to focus on things that are important to me.	porque encorajou-me a centrar em coisas que são realmente importantes para mim.
because it inspired me to think about meaningful issues.	porque inspirou-me a pensar em assuntos com profundo significado.
because it inspired new insights. *	porque inspirou novas ideias.
Thrill	Excitação
because I enjoyed the excitement of it.	porque apreciei a excitação que me fez sentir.
because I liked the adrenaline I got from it.	porque gostei da adrenalina que retirei dessa experiência.
because I enjoyed the thrill of it.	porque apreciei a intensidade do que senti.
because I liked the tension associated with it.	porque gostei da tensão que me fez sentir.
Character engagement	Envolvimento com personagens
because I liked to slip into the role of characters.	porque gostei de encarnar o papel das personagens.
because I liked to live through and share the characters’ experiences.	porque gostei de partilhar experiências dos personagens e vivê-las através deles.
because I liked to feel with characters.	porque gostei de sentir os sentimentos das personagens.
because I identified with the characters’ outlook on life. *	porque identifiquei-me com a visão que os personagens têm da vida.
Vicarious release of emotions	Libertação vicariante das emoções
because it allowed me to experience feelings that I cannot act on in everyday life.	porque permitiu-me sentir emoções que na vida real não posso libertar.
because it allowed me to experience feelings that I normally have to hide in everyday life.	porque permitiu-me experienciar emoções que geralmente tenho de ocultar no dia a dia.
because I could experience feelings that are difficult for me to allow in everyday life.	porque consegui experimentar emoções a que não me posso permitir no dia a dia.
because it allowed me to experience emotions that I avoid in everyday life.	porque permitiu-me experienciar emoções que evito na vida real.
Empathic Sadness	Tristeza empática
because I like to have a good cry.	porque gosto de uma "boa dose de choro."
because I like being moved to tears.	porque gosto de ficar emocionado até às lágrimas.
because I like moments of sadness and poignancy.	porque gosto de momentos tristes e comoventes.
because I liked to be overwhelmed with emotion. *	porque gostei de ser inundado(a) pela emoção.
Social sharing of emotions	Partilha social das emoções
because it stimulated the exchange of comments while watching the movies.	porque ver o filme estimulou a troca de comentários.
because it encouraged me to discuss issues with others.	porque encorajou-me a debater assuntos com outras pessoas.
because it inspired me to talk about the movie with others.	porque inspirou-me a falar com outras pessoas acerca do filme.
because it made me curious to find out how others experienced the movie.	porque fiquei com curiosidade em saber como as pessoas experienciaram o filme.

* items removed from the Portuguese version. Original items in Bartsch [1].
